# Supplementary material for: Metabolomic Insight into Implications of Induction Chemotherapy Followed by Concomitant Chemoradiotherapy in Locally Advanced Head and Neck Cancer
Source: Int J Mol Sci. 2023 Dec 22;25(1):188. doi: 10.3390/ijms25010188 (PMC10779362; doi:10.3390/ijms25010188)
Supplement: Supplementary file 1 [file ijms-25-00188-s001.zip › ijms-2751667-supplementary.pdf]

Supplementary Materials for

Metabolomic Insight into Implications of Induction Chemotherapy Followed by Concomitant Chemoradiotherapy in Locally Advanced Head and Neck Cancer

Łukasz Boguszewicz [1,*], Agata Bieleń [2], Mateusz Ciszek [1], Agnieszka Skorupa [1], Jolanta Mrochem-Kwarciak [3], Krzysztof Składowski [2] and Maria Sokół [1]

[1] Department of Medical Physics, Maria Sklodowska-Curie National Research Institute of Oncology,
Gliwice Branch, 44-102 Gliwice, Poland; mateusz.ciszek@gliwice.nio.gov.pl (M.C.); agnieszka.skorupa@gliwice.nio.gov.pl (A.S.); maria.sokol@gliwice.nio.gov.pl (M.S.)

[2] 1st Radiation and Clinical Oncology Department, Maria Sklodowska-Curie National Research Institute of Oncology, Gliwice Branch, 44-102 Gliwice, Poland; agata.bielen@gliwice.nio.gov.pl (A.B.)

[3] Analytics and Clinical Biochemistry Department, Maria Sklodowska-Curie National Research Institute of Oncology, Gliwice Branch, 44-102 Gliwice, Poland; jolanta.mrochem-kwarciak@gliwice.nio.gov.pl

* Correspondence: lukasz.boguszewicz@gliwice.nio.gov.pl

The characteristics of the acquired spectra, as well as the pulse sequence parameters.

- NOESY (nuclear Overhauser effect spectroscopy)—an overview of all types of molecules;
- CPMG (Carr–Purcell–Meiboom–Gill)—information on only low-molecular-weight metabolites.
- DIFF (diffusion-edited)—mainly macromolecular signals.
- Two-dimensional (2D) JRES (J-resolved)—a visualization of scalar couplings and improved metabolite identification.

Table S1. NMR pulse sequence parameters.

Pulse program	**NOESYGPPR1D**	**CPMGPR1D**	**LEDBPGPPR2S1D**	**JRESGPPRQF**
**TD**	65536	65536	65536	8192
**SW** [ppm]	30	20	30	16.62
**AQ** [sec]	2.73	4.09	2.73	0.62
**D1** [sec]	4	4	4	2
**D8** [sec]	0.01	-	-	-
**D16** [sec]	-	-	0.0002	0.0002
**D20** [sec]	-	0.0003	0.12	-
**D21** [sec]	-	-	0.005	-
**DS**	4	4	4	16
**L4**	-	126	-	-
**NS**	32	64	64	1
**DELTA1** [sec]	-	-	0.11572488	-
**DELTA2** [sec]	-	-	0.004172	-

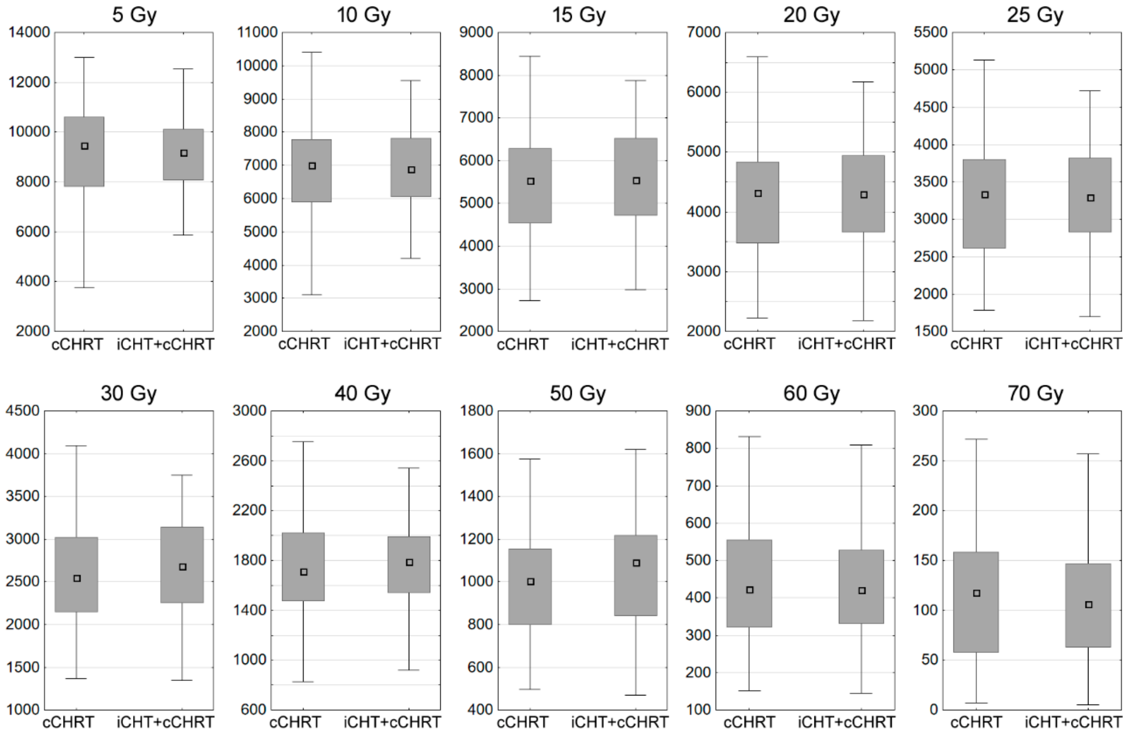

Figure S1. Box plot comparison of the tissue volumes receiving a particular dose of radiation. No statistically significant differences are observed. Point—median; box—25-75 percentile; whiskers—the minimum and maximum values.
